# Optimising Genomic Selection in Wheat: Effect of Marker Density, Population Size and Population Structure on Prediction Accuracy

**DOI:** 10.1534/g3.118.200311

**Published:** 2018-07-03

**Authors:** Adam Norman, Julian Taylor, James Edwards, Haydn Kuchel

**Affiliations:** School of Agriculture, Food & Wine, University of Adelaide

**Keywords:** Genomic prediction, Wheat breeding Marker density, Training set design, GenPred, Shared Data Resources, Genomic Selection

## Abstract

Genomic selection applied to plant breeding enables earlier estimates of a line’s performance and significant reductions in generation interval. Several factors affecting prediction accuracy should be well understood if breeders are to harness genomic selection to its full potential. We used a panel of 10,375 bread wheat (*Triticum aestivum*) lines genotyped with 18,101 SNP markers to investigate the effect and interaction of training set size, population structure and marker density on genomic prediction accuracy. Through assessing the effect of training set size we showed the rate at which prediction accuracy increases is slower beyond approximately 2,000 lines. The structure of the panel was assessed via principal component analysis and K-means clustering, and its effect on prediction accuracy was examined through a novel cross-validation analysis according to the K-means clusters and breeding cohorts. Here we showed that accuracy can be improved by increasing the diversity within the training set, particularly when relatedness between training and validation sets is low. The breeding cohort analysis revealed that traits with higher selection pressure (lower allelic diversity) can be more accurately predicted by including several previous cohorts in the training set. The effect of marker density and its interaction with population structure was assessed for marker subsets containing between 100 and 17,181 markers. This analysis showed that response to increased marker density is largest when using a diverse training set to predict between poorly related material. These findings represent a significant resource for plant breeders and contribute to the collective knowledge on the optimal structure of calibration panels for genomic prediction.

For breeders to make the best use of genomic selection, several factors influencing genomic prediction accuracy should be well understood from empirical breeding germplasm datasets in order to optimize rates of genetic gain. Also, before breeding programs divert finite resources toward the implementation of genomic selection, a number of potentially derailing features of diversity based genetic analysis deserve further attention.

Genomic selection involves estimating a large number of marker effects using a set of training lines, and then using these to predict the value of a separate set of lines (Meuwissen *et al.*, 2001). Three major factors that affect the accuracy at which lines can be predicted are training set size, marker density, and population structure, which have been studied previously in wheat populations up to 8,416 lines in size (Nakaya and Isobe 2012; Crossa *et al.*, 2016; He *et al.*, 2017). Larger training sets were shown to increase prediction accuracy within bi-parental populations by Heffner *et al.* (2011a), where training sets consisted of 24 to 96 lines, and also in multifamily populations by Heffner *et al.* (2011b), where training sets ranged from 96 to 288 lines in size. This result was corroborated by Isidro *et al.* (2015) and Michel *et al.* (2017) where training sets up to 300 lines in size were tested. Training sets consisting of up to 3,052 lines have been used in other studies, but not to directly investigate the effect of training set size. Larger training sets give higher prediction accuracy as increased sample size reduces bias and decreases the variance of marker effect estimates (Liu *et al.*, 2011; de los Campos *et al.*, 2013). Of the studies investigating the effect of training set size, none reached the point where further increases in size would not continue to increase prediction accuracy. Here we address this question using uniquely large training sets (n = 8,300). This research therefore provides the most relevant results to large scale breeding programs which typically work with tens of thousands of lines.

Another factor for breeding programs to consider is the required marker density. Prediction accuracy increases with marker density due to more quantitative trait loci (QTL) being in LD with a marker (Heffner *et al.*, 2009; Desta and Ortiz 2014). Solberg *et al.* (2008) showed using simulated data that increasing single nucleotide polymorphism (SNP) density from one to eight SNP per cM resulted in a 25% increase in prediction accuracy. Heffner *et al.* (2011b) used a multifamily wheat dataset to show a 10% increase in prediction accuracy was achieved when moving from 192 to 1,158 markers. However, most of this increase occurred from 192 to 384 markers, indicating that the response to increased marker density would eventually reach a plateau (de los Campos *et al.*, 2013). The point at which this plateau occurs is determined by the genetic diversity within the population, and the relatedness between the training and prediction sets. Hickey *et al.* (2014) showed in a maize simulation study that fewer markers are required when there is high relatedness between training and prediction sets, as they share long haplotype effects and large linkage blocks. The study also found that increasing the size and diversity of the training set was only beneficial when using a large number of markers. Heffner *et al.* (2011a) investigated the response of prediction accuracy to marker density using bi-parental wheat populations, and found a positive response up to 256 markers but a decrease when increasing to 384. As explained by Hickey *et al.* (2014), large numbers of markers can result in the model being overfitted, where non-genetic effects are attributed to the markers. While this improves the model fit, it decreases the accuracy of predicting independent data sets which do not share the non-genetic effects (Jannink *et al.*, 2010). Previous studies have investigated the required marker density in wheat using small empirical datasets of up to 1,158 markers, while other species have been studied using simulated datasets. The current study uses a much larger empirical dataset to extend previous findings into the range where responses can plateau.

Discrete groups of lines with contrasting origin often have differences in allele frequency (population structure) due to selection or parentage (founder effects) (Isidro *et al.*, 2015). This can be problematic as differences in observed phenotypic performance between the two groups may be associated with the markers differing in allele frequency, regardless of whether they are linked to the QTL responsible for the trait variance (Price *et al.*, 2010). The underlying structure of a population is commonly assessed and accounted for using principal component analysis (PCA) of the complete genetic marker set (Patterson *et al.*, 2006; Bentley *et al.*, 2014; Daetwyler *et al.*, 2014). This is an effective method for identifying and visualizing the genetic structure of diverse germplasm panels.

The extent and nature of genetic structure within and across training and validation sets influences the achievable prediction accuracy, and is therefore of interest to breeders when designing training sets. When the training set contains lines closely related to those being predicted, accuracy is higher due to shared long haplotype effects (Daetwyler *et al.*, 2013). Ben Hassen *et al.* (2018) recently observed this relationship in a small rice germplasm set. However, these large linkage blocks are quickly broken up by recombination events, and so crossing cycles can rapidly decrease prediction accuracy (Hickey *et al.*, 2014). If marker density is adequate, increased diversity in the training set will lead to calibration by linkage disequilibrium where short haplotype effects are exploited; this is more stable over multiple generations of crossing (Hickey *et al.*, 2014). However, distant relationships increase noise and bias in the genomic relationship matrix, which in turn reduces the power of prediction (Lund *et al.*, 2016). This study uses multiple breeding cohorts from a commercial breeding program in a unique cross-validation design to investigate the interaction of these opposing effects in an applied scenario, which will inform breeders on optimal training set design.

In this research we study the optimal design of genomic selection training sets by using a panel of 10,375 wheat lines to investigate the effect that training set size, marker density, and genetic structure have on genomic prediction accuracy. We also examine the interaction between marker density and population structure.

## Materials and Methods

### Plant material and associated data

This study utilizes an association panel of 10,375 bread wheat lines, sourced from preliminary and advanced yield testing programs of Australian Grain Technologies Pty Ltd (AGT). The panel was phenotyped in a dedicated field trial at Roseworthy, South Australia (-34.52, 138.69) in the 2014 growing season. We studied data from a single site in order to remove the potentially confounding effect of genotype by environment interaction (GxE). As described in Norman *et al.* (2017), the trial was sown as a non-replicated randomized design with repeated grid checks (1 check per 11 plots), as the large number of lines made a replicated trial logistically infeasible. Dimensions of the trial were 476 rows by 24 ranges, and plot size was 3m^2^. The trial was managed according to best local practice which included fertilizer applications to maximize grain yield and grain quality, and fungicide applications to control disease. Grain yield was measured with a machine harvester and thousand kernel weight (TKW) through image analysis. Both glaucousness and relative maturity were assessed visually, glaucousness on a 1-9 scale (1 = low expression) and relative maturity using the Zadoks scale (Zadoks *et al.*, 1974). These four traits were selected for the current study as they display sufficient phenotypic variation, represent varying levels of genetic control, and experience different selection pressure in a breeding program. Glaucousness has simple genetic control (Bennett *et al.*, 2012a; Norman *et al.*, 2017) and was not actively selected for in this breeding program. Maturity is predominantly controlled by several large effect genes (Snape *et al.*, 2001; Cane *et al.*, 2013) and is selected for mid range performance suitable for the Australian environment. TKW is quantitative (Huang *et al.*, 2006; Sun *et al.*, 2009; Bennett *et al.*, 2012b), and lines are heavily selected to perform above a threshold. Grain yield is a highly complex trait (Kuchel *et al.*, 2007; Bennett *et al.*, 2012b; Maphosa *et al.*, 2014) and lines are strongly selected to yield as high as possible.

Marker genotyping was performed using a custom Axiom™ Affymetrix array containing 18,101 single nucleotide polymorphism (SNP) markers. Markers with minor allele frequency (MAF) lower than 0.01 were removed. Further details on the development of the genotyping platform and preparation of the marker data are provided in Norman *et al.* (2017).

### Statistical modeling

#### One step genomic prediction model:

In this research we followed the statistical modeling approach similar to Norman *et al.* (2017). Initially, the phenotypic data from the full Roseworthy trial as well as the complete genotypic marker data was used to form a one-step genomic prediction linear mixed model. Let $y=\left(y_{1},\ldots ,y_{n}\right)$ be a vector of trait observations then the linear mixed model had the form

$$y=X\tau +\mathrm{Zu}+Z_{g}g+e$$ (1)

where τ was a vector of fixed effects with associated design matrix X, and contains an intercept and coefficients for covariates in X explaining potential trends or known environmental anomalies across the layout of the trial. Extraneous non-genetic variation due to the experimental design such as blocks were captured using random effects u with design matrix Z where the effects were assumed to be distributed $u∼N\left(0,\sigma_{u}^{2}I\right).$ To ensure dependence between trait observations was appropriately modeled, the residual error, **e,** was assumed to be distributed $e∼N\left(0,\sigma^{2}R\right)$ where $R=R_{r}\left(\rho_{r}\right)⊗R_{c}\left(\rho_{c}\right)$ was parameterised as a separable AR1 ⊗ AR1 (AR1 = auto-regressive of order 1) correlation structure in the row and column dimensions of the experimental layout (Gilmour *et al.*, 1997). In (1) the $n_{g}$ length vector of total genetic effects g were defined by the genetic model

g=a+p (2)

where a and p were the additive and residual genetic effects respectively with joint distribution

$$\left[\begin{array}{c} a\\ p \end{array}\right]∼N\left(\left[\begin{array}{c} 0\\ 0 \end{array}\right],\left[\begin{array}{cc} \sigma_{a}^{2}K & 0\\ 0 & \sigma_{p}^{2}I \end{array}\right]\right)$$

Here, $K=MM^{T}/s$ where M is the complete marker matrix and *s* is a scaling constant defined by $s=\left({\sum^{\text{​}}}_{j=1}^{n_{g}}d_{jj}\right)/n_{g}$ where $d_{jj}$ is the *j*th diagonal element of $MM^{T}$ (Forni *et al.*, 2011). The matrix K is known as the additive relationship or kinship matrix (VanRaden 2008) and can be viewed as a full rank variance matrix detailing the additive connectivity between the genotyped lines. The constant *s* ensures the genetic variance parameters $\sigma_{a}^{2}$ and $\sigma_{p}^{2}$ are numerically comparable and interpretable.

Parameter estimation in the one-step genomic prediction linear mixed model (1) was achieved through an iterative algorithm. Best linear unbiased estimators (BLUEs) of the fixed effects and best linear unbiased predictions (BLUPs) of the random effects were obtained from solutions to the mixed model equations (MMEs) (Henderson 1953). Estimates of the variance parameters are then obtained through an average information algorithm (Gilmour *et al.*, 1995) implemented through maximizing the residual maximum likelihood (REML) derived in Patterson and Thompson (1971). From these solutions the genomic best linear unbiased predictions (GBLUPs) of the additive genetic effects **a** can be written as

$$\tilde{a}=\sigma_{a}^{2}KZ_{g}^{T}\mathrm{Py}$$ (3)

where $P=H^{-1}-H^{-1}X{\left(X^{T}H^{-1}X\right)}^{-1}X^{T}H^{-1}$ and $H=\text{var}\left(y\right)=\sigma^{2}R+\sigma_{u}^{2}ZZ^{T}+\sigma_{a}^{2}Z_{g}KZ_{g}^{T}+\sigma_{p}^{2}Z_{g}Z_{g}^{T}.$ These GBLUPs $\tilde{a}$ represent the relative genetic merit of the lines and are commonly called estimated breeding values.

#### Cross validation:

For each cross-validation scenario conducted, training data sets were created by setting the validation set records from the phenotypic data to missing and appropriately subsetting the genetic marker data to include training set lines only. A training set model was fitted using an adaptation of the linear mixed model defined in (1) with non-genetic parameters fixed at their estimates from the full model. Marker effects were then predicted using the methods described in Norman *et al.* (2017), namely

$${\tilde{q}}_{t}=M_{t}^{\text{T}}K_{t}^{-1}{\tilde{a}}_{t}$$ (4)

where $M_{t}$ and $K_{t}$ were the genetic marker data and additive relationship matrix respectively for the training set of lines and ${\tilde{a}}_{t}$ were GBLUPs for training lines calculated using (3). Genomic predictions for lines in the validation set were then determined using

$${\tilde{a}}_{v}=M_{v}{\tilde{q}}_{t}$$ (5)

where $M_{v}$ is the genetic marker data for the validation set and ${\tilde{q}}_{t}$ is defined in (4).

For cross-validation scenarios in section 2.6 where the number of markers is reduced below the number of lines used in the training set, the model (1) cannot be used due to rank deficiency in the relationship matrix. Consequently, an alternative formulation was adopted for the genetic effects defined in (2), namely

$$g_{t}=M_{t}^{*}q_{t}+p_{t}$$ (6)

where $M_{t}^{*}$ is the genetic marker data with reduced numbers of markers for the training set, $q_{t}$ represents a vector of marker effects with assumed distribution $q_{t}∼N\left(0,\sigma_{a}^{2}I\right)$ and $p_{t}$ are the residual genetic effects defined in (2). The iterative estimation algorithm proceeds similarly to the previous section and marker effect predictions for the training set were determined directly using

$${\tilde{q}}_{t}=\sigma_{a}^{2}M_{t}^{*T}Z_{g}^{T}\mathrm{Py}$$

GBLUPs of the additive effects for the validation lines were then immediately determined using an analogous equation to (5), namely ${\tilde{a}}_{v}=M_{v}^{*}{\tilde{q}}_{t}$.

#### Computations:

All statistical analysis was carried out in the R Statistical Computing Environment (R Core Team 2017). Linear mixed models were fitted using the flexible linear mixed modeling package ASReml-R (Butler *et al.*, 2009) available as an R package and downloadable from *www.vsni.co.uk/software/asreml*.

### Impact of training set size on prediction accuracy

The effect of training set size on genomic prediction accuracy was assessed through an extended five-fold cross-validation analysis. First, the full panel was randomly divided into five folds each containing 2,075 lines. Four of these folds acted as a training set (8,300 lines) which was used to predict the remaining fold (validation set). The training set was then randomly sampled to sizes of 250, 500, 1,000, 2,000, 3,000, 4,000, 5,000, 6,000 and 7,000, where each acted as a training set to predict that fold’s validation set which remained at a fixed size of 2,075 lines. These subsets were sampled without replacement resulting in varying levels of replication for the different sizes. Within each fold there were 33 reps at 250, 16 of 500, 8 of 1,000, 4 of 2,000, 2 of 3,000, 2 of 4,000, and 1 of 5,000 and above. All training models were fitted according to (1) where marker effects were then calculated by (4), and used to form genomic predictions of lines in the validation set according to (5). All training sets within each fold were used to predict the same validation set. Relative prediction accuracies were calculated by correlating the genomic predictions to the corresponding additive GBLUP values from the full data set model. For the remainder of this paper, the term prediction accuracy is used to describe the capacity of the comprised training sets to predict line performance as described by the maximal model.

### Impact of population structure on prediction accuracy

To investigate how genomic prediction training sets can be optimally designed, the panel was partitioned using two different approaches for the purposes of training and cross-validation. In the first method, K-means clustering was used to partition based on genetic similarity. This was used as a surrogate for assessing calibration within and between germplasm pool (breeding program). In the second method, the germplasm was partitioned by cross-year to examine the effect on prediction accuracy of including multiple historical ‘breeding cohorts’ (historical lines/data). Online Resource 1 details which lines belong to each cluster and breeding cohort.

#### Impact of underlying population structure:

**Figure 1:**
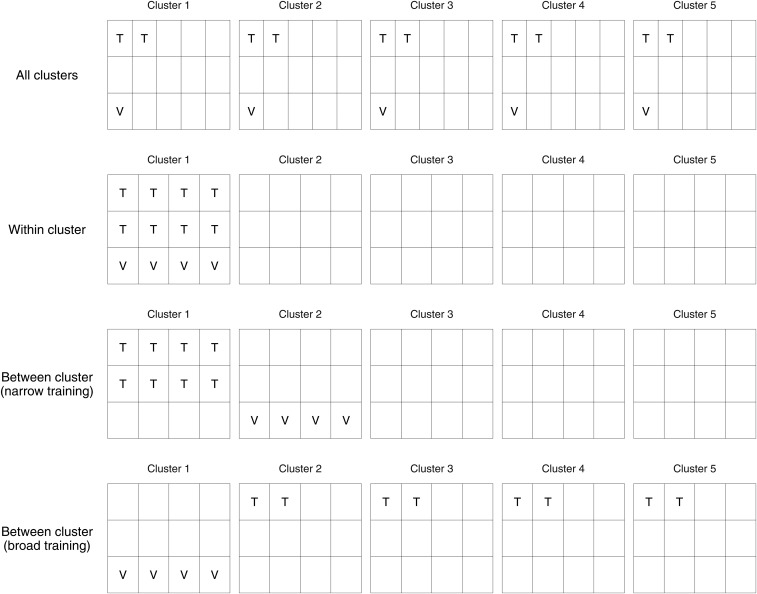
Description of the four cross-validation designs used to assess the impact of underlying population structure. The partitions within each cluster were formed by randomly sampling without replacement. Replication was achieved by rotating partitions within each design to provide all combinations of partitions and clusters. All designs had consistent training and validation set sizes of 1,000 and 500 respectively.

K-means clustering was performed on a marker based genetic dissimilarity matrix using the K-means functionality inside the R statistical computing environment (R Core Team 2017). The sum of squares within clusters was assessed when setting the number of clusters between 2 and 50, which showed the variance plateaus when there were more than five. The number of clusters was therefore set at five. In order to achieve clusters of equal size, 1,500 lines were randomly selected from each to be used in the cross-validation analysis. With these 7,500 lines, four cross-validation designs (detailed in Figure 1) were then used to achieve i) equal representation of all clusters in both the training and validation sets (‘all clusters’), ii) representing the same cluster in both the training and validation set (‘within cluster’), iii) representing one cluster in the training set and one different cluster in the validation set (‘between cluster - narrow training’), and iv) representing four clusters in the training set and the remaining one cluster in the validation set (‘between cluster - broad training’). Within each cluster, lines were randomly sampled without replacement to produce subsets. This allowed all training sets in each of the four designs to contain 1,000 lines, and all validation sets to contain 500 lines. In each design these subsets were rotated to all possible combinations in order to provide replication. All training models were fitted according to (1) where marker effects were then calculated by (4), and used to form genomic predictions of lines in the validation set according to (5). Prediction accuracies were calculated by correlating the genomic predictions to the corresponding additive GBLUP values from the full data set model.

#### Impact of breeding cohort:

Here, lines from four different breeding cohorts were selected from the PYT-South subset of breeding lines. The cohorts were randomly selected from the second yield testing stage of the south breeding program from years 2010 to 2013, and each cohort contained 996 lines. Three cross-validation designs were used to assess i) one cohort year (training set) used to predict the following cohort year (validation set), ii) two cohort years (training set) used to predict the following cohort year (validation set), and iii) three cohort years (training set) used to predict the following cohort year (validation set). As in the K-means clustering method, lines were randomly sampled without replacement within each cohort year to produce subsets. This allowed all training and validation sets in each of the three designs to contain 996 lines. In design ii) the training sets were made up of one 498 line subset from each of the two cohort years, and in design iii) they consisted of one 332 line subset from each of the three cohort years. Cross-validations were performed according to the same methods used in the K-means clustering method.

### Marker density analysis

Marker subsets of varying size (100, 500, 1,000, 3,000, 5,000, 10,000, 13,639 and 17,181) were selected in order to assess the effect of marker density on prediction accuracy, and its interaction with population structure. The 13,639 markers on the consensus map from Norman *et al.* (2017) were selected as the first subset, from which markers for the lower densities were selected with the criteria of being evenly distributed on the genome, as well as having high minor allele frequency (MAF). To achieve this, markers were first allocated into linkage map bins of varying size for each target density, and those with the highest MAF within each bin were selected. Table 1 summarizes each marker subset and genetic maps of each subset are plotted in Online Resource 2. Online Resource 3 details which markers belonged to each subset.

**Table 1:** Summary of the marker selections using the consensus map

Number of markers	Unique map positions	Markers per map position	Mean interval*^a^*	Mean MAF*^b^*
100	100	1.00	31.2	0.49
500	500	1.00	6.25	0.44
1000	1000	1.00	3.12	0.40
3000	3000	1.00	1.04	0.34
5000	4580	1.09	0.68	0.32
10000	4590	2.18	0.68	0.29
13639	4593	2.97	0.68	0.26

a Mean interval (cM) between unique map positions.

b Mean minor allele frequency across the full panel.

#### Random five-fold cross validation:

The effect of marker density on prediction accuracy was assessed with random five-fold cross validation, where training sets consisted of 8,300 lines and validation sets 2,075 lines. The cross validation was repeated for each marker density. Training models for marker densities containing fewer markers than lines were fitted according to (6) where marker effects were determined directly. For densities containing more markers than lines, training models were fitted according to (1) and marker effects were then calculated through (4). Marker effects from either method were then used to formulate genomic predictions of lines in the training set according to (5), and prediction accuracies were calculated by correlating the predictions to additive GBLUP values from the full model.

#### K-means clustering:

The response of prediction accuracy to marker density was assessed in different population structures by repeating the K-means clustering method for each marker density. As in section 2.6.1, training models for densities containing fewer markers than lines were fitted according to (6), and those containing more markers than lines were fitted according to (1). Genomic predictions were calculated according to (5), and correlated to GBLUP values from the full model to determine prediction accuracy.

### Data availability

File S1 specifies the breeding cohorts used for analysis. File S2 contains genetic map plots of each marker subset. File S3 specifies which markers were included in each subset, and the genetic map position of each marker. File S4 contains all genetic marker data, and file S5 contains all phenotype data. Supplemental material available at Figshare: https://figshare.com/s/287c2c7f1623008487a5.

## Results

### Impact of training set size on prediction accuracy

**Figure 2:**
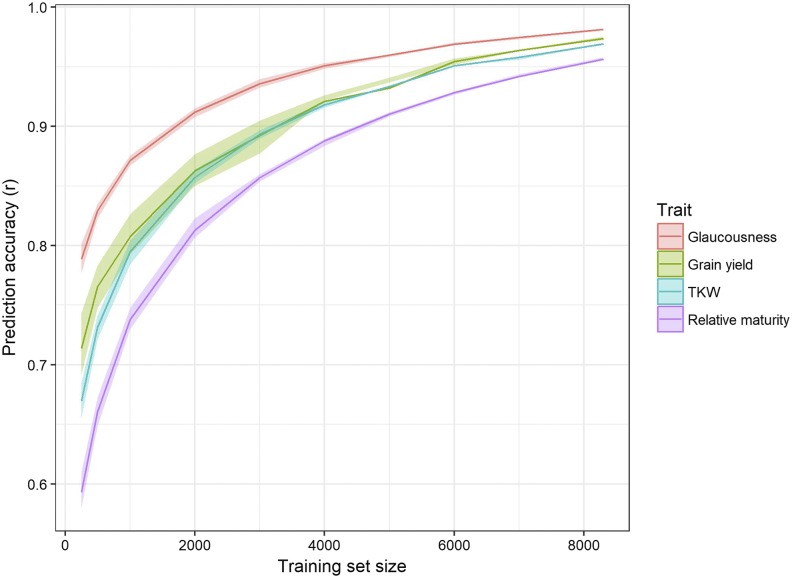
Genomic prediction accuracies from five-fold random cross-validation with varying training set sizes. Shading represents upper and lower quartiles. Prediction accuracy is defined as the correlation between genomic predictions of the validation set and their corresponding additive GBLUP values from the maximal model. TKW represents thousand kernel weight.

Figure 2 details the effect of training set size on genomic prediction accuracy for the four traits analyzed. A similar trend was observed at each trait with accuracy increasing substantially from training set size of 250 to 2,000. A correlation with the maximal model of 0.95 was achieved with training set sizes of between 3,950 and 7,650 (for traits glaucousness and relative maturity respectively). Glaucousness was the most accurate trait at all sizes, and maturity the least. The difference in accuracy between traits was more pronounced at smaller training set sizes (0.59 to 0.79 at size 250, 0.96 to 0.98 at size 8,300). Grain yield showed the most variation between replications of each training set size (indicated by the shading of upper and lower quartiles), and glaucousness the least.

### Impact of population structure on prediction accuracy

**Figure 3:**
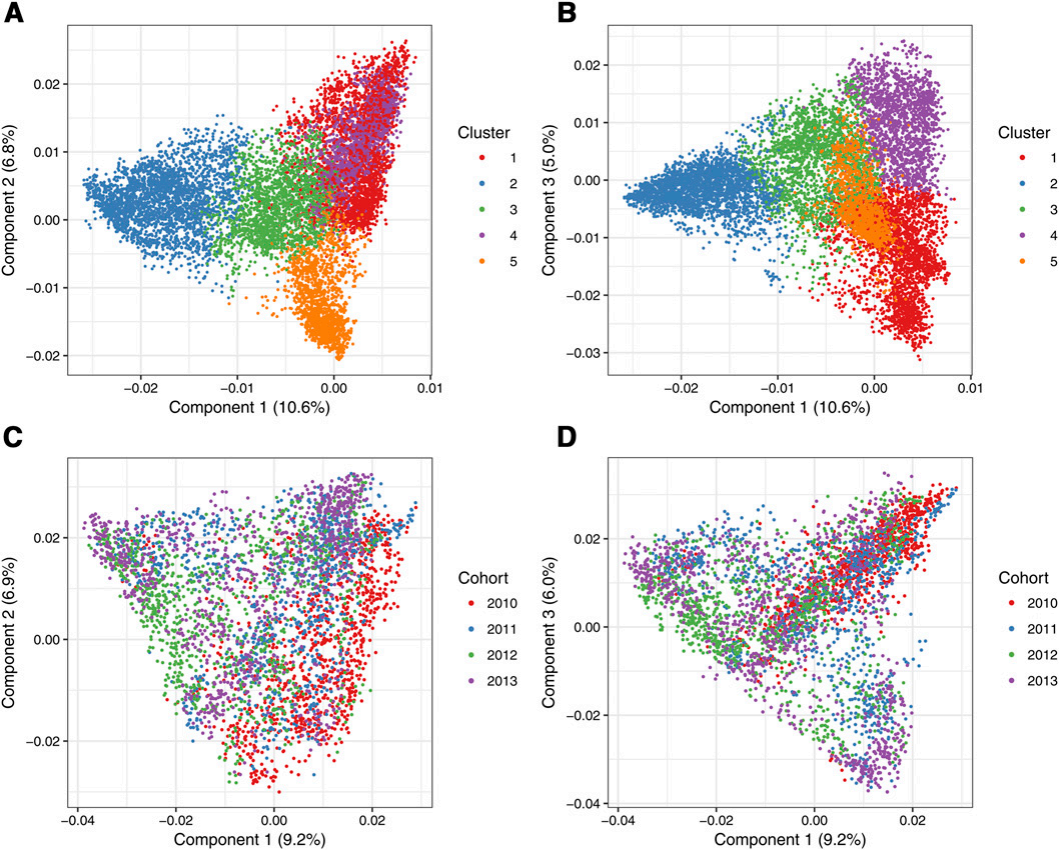
Pairwise plots of components from two principal component analyses (PCA). A First and second components of the PCA performed on lines included in the K-means clustering method, with lines colored according to which cluster they belonged. B First and third components of the PCA performed on lines included in the K-means clustering method, with lines colored according to which cluster they belonged. C First and second components of the PCA performed on lines included in the breeding cohort method, with lines colored according to which cohort they belonged. D First and third components of the PCA performed on lines included in the breeding cohort method, with lines colored according to which cohort they belonged.

Figure 3 details the structure of lines included in each of the population structure analyses. Sub-plots **A** and **B** display components one and two, and one and three respectively from a PCA performed on the lines included in the K-means cluster analysis. Sub-plots **C** and **D** represent similar plots from a PCA performed on the lines included in the breeding cohort analysis, where lines are colored according to their cohort year. There is a clear distinction between the K-means clusters, while the genetic dissimilarity between the breeding cohorts is less pronounced.

#### Impact of underlying population structure:

**Figure 4:**
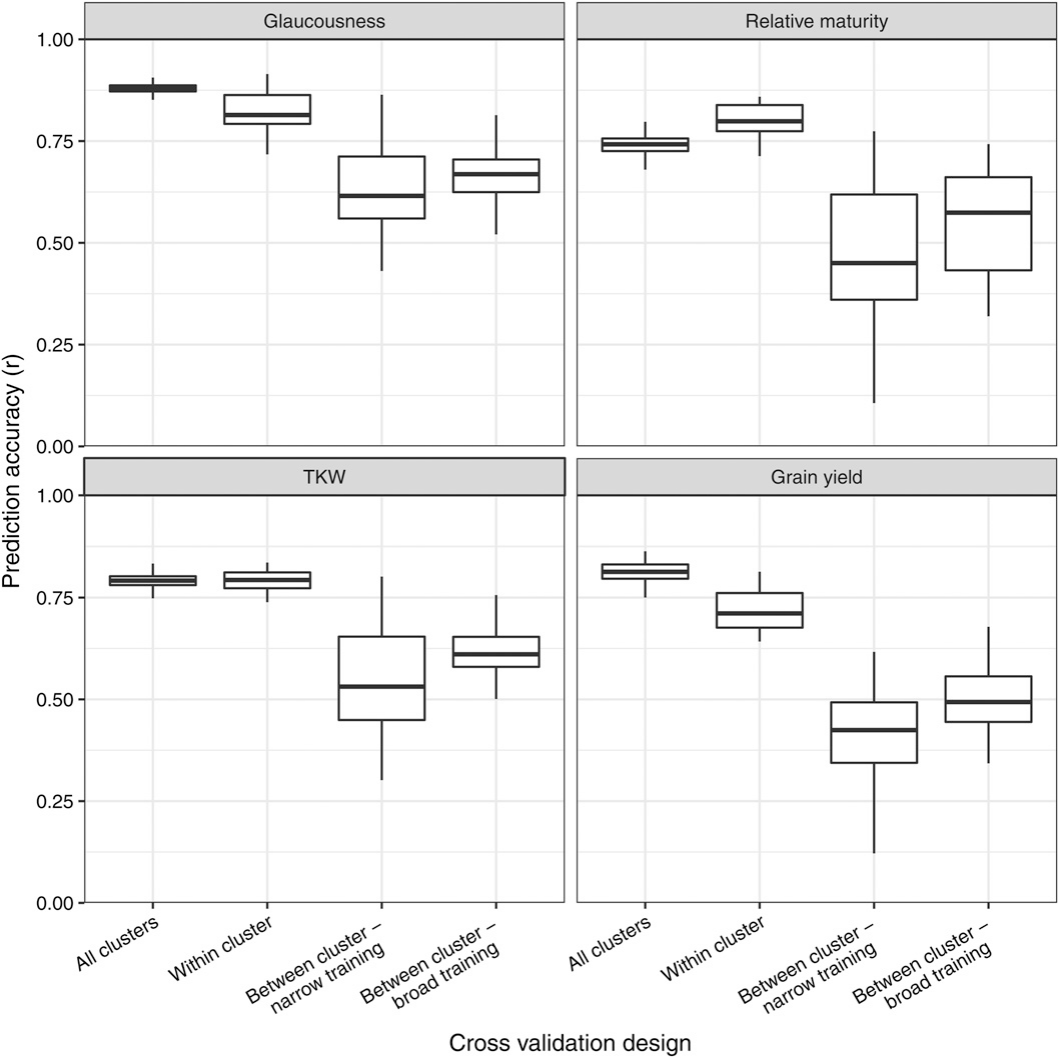
Boxplots showing prediction accuracies from the K-means clustering method for each category of training and validation set combinations, detailed in section 2.5.1. Prediction accuracy was calculated by correlating predictions of the validation set to the corresponding additive GBLUP values from the full model with all lines included. TKW represents thousand kernel weight.

Figure 4 summarizes prediction accuracies from the K-means clustering method of assessing population structure impacts on prediction accuracy. ‘All clusters’ and ‘within cluster’ accuracies were similarly high for glaucousness and grain size, whereas for grain yield ‘all clusters’ was slightly higher and for relative maturity slightly lower. For all traits, predicting between cluster with a broad training set was more accurate than predicting between cluster with a narrow training set, but both were significantly less accurate than ‘all clusters’ and ‘within cluster’.

#### Impact of breeding cohort:

**Figure 5:**
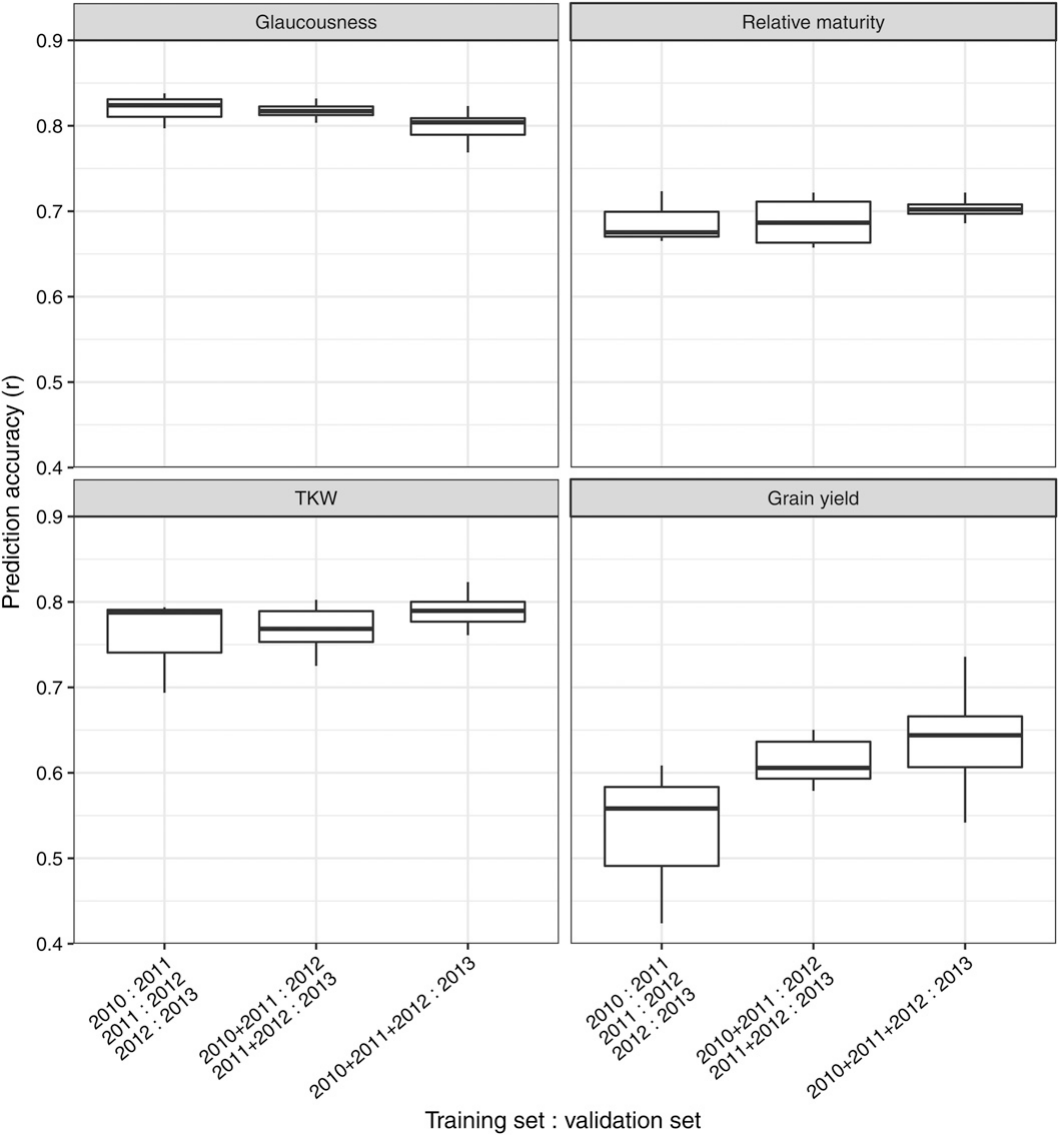
Boxplots summarizing the prediction accuracies from the breeding cohort method, as detailed in section 2.5.2. At each trait the first boxplot represents one cohort year used as a training set to predict the subsequent cohort year (validation set). The second represents two consecutive cohort years used as training to predict the subsequent cohort year, and third represents three consecutive cohort years used to predict the subsequent.

Figure 5 presents prediction accuracies from the breeding cohort method of assessing the impact of population structure on prediction accuracy. This shows that as more cohort years were represented in the training set, prediction accuracy increased significantly for grain yield, and slightly for relative maturity. Glaucousness and TKW however, had relatively stable prediction accuracy regardless of how many cohort years were represented in the training set. Prediction accuracies were highest for TKW and glaucousness, with relatively maturity being slightly lower and grain yield lower again.

### Impact of marker density on prediction accuracy

Table 1 summarizes each marker selection using the consensus map to calculate unique positions, markers per map position and mean interval. This shows only a slight increase in the number of map positions at selections containing more than 5,000 markers. Therefore, at the selections with more than 5,000 markers, the mean position interval plateaus off and markers per map position increases. The mean MAF of the markers at each selection starts very high at 0.49 for the 100 marker selection, and steadily decreases to 0.26 for the 13,639 selection.

**Figure 6:**
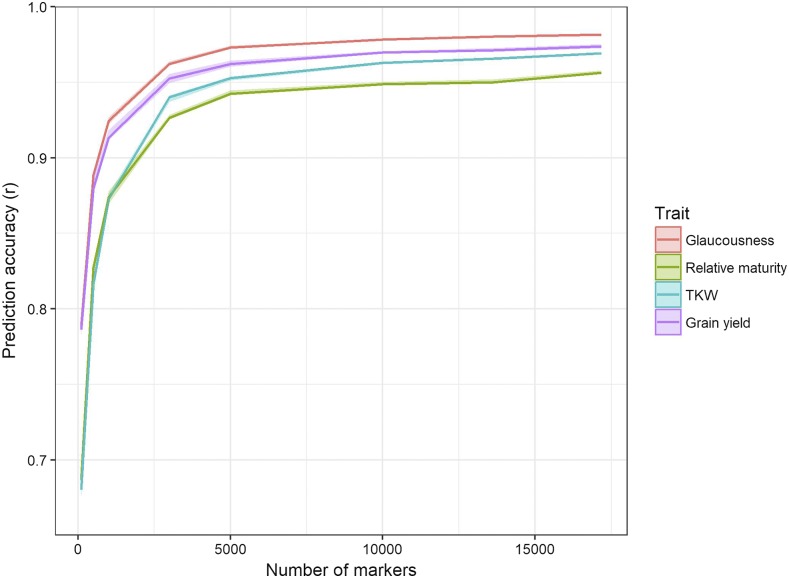
Plot showing the effect of marker density on prediction accuracy for each trait. Prediction accuracy was assessed by performing random five-fold cross-validation for each selection of markers, and correlating predictions of the validation set to the corresponding additive GBLUP values from the full model with all lines included. Marker subsets were selected to be evenly distributed over the genome and to have high minor allele frequency.

The effect of marker density on prediction accuracy was assessed in the first instance through random five-fold cross validation, the results of which are summarized in Figure 6. All four traits showed a sharp increase in accuracy before reaching a plateau at approximately 5,000 markers, with only a marginal increase in prediction accuracy when increasing from 5,000 to 17,181 markers. All traits showed the highest prediction accuracy when all available markers were used. Glaucousness, relative maturity and grain yield all had similar response curves, but TKW had a more pronounced increase in accuracy with marker number, particularly when increasing from 1,000 to 3,000 markers.

### Effect of interaction Between marker density and population structure on prediction accuracy

**Figure 7:**
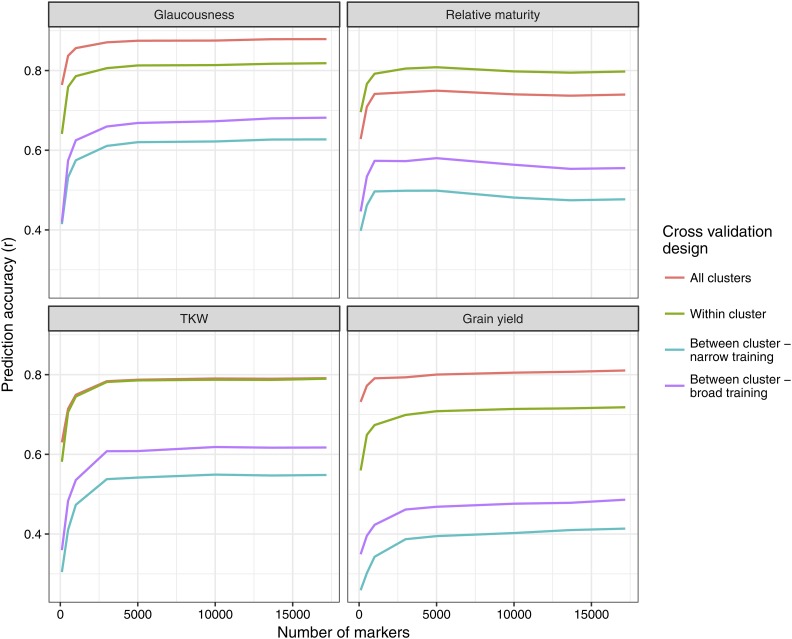
Plots showing the interactive response of prediction accuracy to marker density and population structure. The K-means clustering method detailed in section 2.5.1 was repeated for each selection of markers. Marker subsets were selected to be evenly distributed over the genome and to have high minor allele frequency.

The K-means clustering analysis was repeated for each marker density in order to investigate the interaction between population structure and marker density (Figure 7). Similar to the five-fold cross validation analysis, prediction accuracies increased sharply up to approximately 3,000 markers before plateauing. Similar responses were observed for ‘all clusters’ and ‘within cluster’ prediction structures across all traits. Between cluster prediction saw greater response to increased marker density, particularly with broad training when increasing from 100 to 1,000 markers. Relative maturity saw a slight decrease in prediction accuracy when marker number was increased beyond 5,000.

## Discussion

If plant breeders are to effectively apply genomic selection in their breeding programs, they require a sound understanding of factors affecting prediction accuracy in large scale germplasm datasets. In the present study we utilized a panel of 10,375 lines sourced from an active breeding program to investigate the effect and interaction of training set size, population structure and marker density on prediction accuracy. The findings presented here will assist breeders in optimizing their programs, allowing them to make the most effective and efficient use of their resources when implementing genomic selection.

### Effect of training set size on prediction accuracy

An important factor influencing genomic prediction accuracy is the size of the training set used to develop the prediction calibration (Nakaya and Isobe 2012). However, research questions pertaining to this have previously proven difficult to address, as the large number of lines required to locate the point of diminishing returns has an often prohibitively high cost of genotyping. The data set analyzed here provides a unique opportunity to investigate the effect of population size on prediction accuracy in bread wheat. Prediction accuracy increased substantially when the training set size was increased from 250 lines to approximately 2,000, after which the rate of increase slowed. While an acceptable prediction accuracy would be determined by the breeder on a case by case basis, if we look at an accuracy of 0.95 as an example, this is achieved at a training set size of 3,930 and 7,450 for glaucousness and relative maturity respectively. This result confirms previous findings from smaller populations (Heffner *et al.*, 2011a, b; Isidro *et al.*, 2015), and extends the relationship to larger training sets showing there is a point at which accuracy begins to plateau in response to increased training set size. Plant breeders should take this result into account when weighing up the benefit of including additional lines in a training set. While there were differences between traits in the level of accuracy achieved, the trend in response to training set size was consistent for all traits despite their differences in genetic complexity. This suggests that response in prediction accuracy to training set size is not dependent on the complexity and genetic architecture of the trait.

The difference in prediction accuracy between traits was more pronounced at smaller training set sizes. This was also driven by the genetic complexity of the trait, as more lines are needed to provide the high number of allelic observations required to accurately predict small effect QTL (Gilmour 2007). Prediction accuracies in this analysis varied more within the smaller training set sizes than the large, particularly for grain yield. This indicates population structure was present and the variation in accuracy was likely caused by the presence or absence of highly related lines across training and validation sets. (Poland *et al.*, 2012). In the next section we investigate how the relatedness between training and validation sets affects the resultant accuracy.

### Effect of population structure on prediction accuracy

K-means clustering produced five genetically distinct clusters, which is demonstrated in Figure 3. Prediction accuracy within and between clusters was tested using structured cross-validation with training sets containing 1,000 lines and validation sets containing 500 lines. The breeding cohorts were less distinct as they were all sourced from the southern breeding program. The accuracy of predicting one cohort using a training set sourced from one, two or three prior cohorts was tested using training and validation sets of the same size as those in the K-means clustering method. This unique assessment is representative of how genomic prediction would be applied in a commercial breeding program.

In the K-means cluster method, ‘all clusters’ and ‘within cluster’ prediction accuracies were similar for glaucousness and TKW. The training sets of both prediction structures directly represent the clusters in their respective validation set, the only difference being that ‘all clusters’ uses all five clusters whereas ‘within cluster’ uses just one. This result therefore suggests the broadness of the training and validation sets has little effect on prediction accuracy when the training set contains at least some lines that are highly representative of those being predicted. For relative maturity however, ‘within cluster’ prediction accuracy was slightly higher than ‘all clusters’, and the reverse was observed for grain yield. There are several large effect photoperiod and vernalisation genes that control maturity (Snape *et al.*, 2001; Cane *et al.*, 2013), and the predominating genes differ between clusters (data not shown). The higher accuracy when predicting maturity within cluster was therefore likely to be caused by the key large effect genes having greater representation in the training set. For grain yield on the other hand, the increased diversity was beneficial as ‘all clusters’ showed higher prediction accuracy than ‘within cluster’. This is because there was more and comparable phenotypic diversity represented within both the training and validation sets for ‘all clusters’.

For all traits, predicting a single cluster using a broad training set produced higher accuracies than predicting with narrow training, but was substantially less accurate than ‘within cluster’ and ‘all clusters’. This shows that prediction accuracy is significantly higher when the training set contains close relatives of lines in the validation set, but accuracy can also be increased by including more genetic diversity in the training set. Breeders should therefore design genetically diverse training sets that are highly related to the prediction set in order to maximize genetic response to genomic selection. This is corroborated by the results of the breeding cohort cross-validation, where prediction accuracy was improved for grain yield and relative maturity by including more cohort years (and therefore more diversity) in the training set. With increased genetic diversity and high SNP density, the training set can better capture short haplotype effects that are relevant to the validation set. This type of calibration is based on short haplotype effects and linkage disequilibrium information, and is suggested by Hickey *et al.* (2014) to be less susceptible to breaking down after multiple breeding cycles.

The breeding cohort analysis is the most representative of how genomic selection would be applied in a breeding program, predicting the current cohort using previous cohorts. The increase in prediction accuracy with more cohorts in the training set was most pronounced for grain yield, and supports previous findings in rye (Auinger *et al.*, 2016). Muir (2007) observed through simulation of animal breeding that continued selection over multiple generations eventually reduced prediction accuracy. The difference between that study and the present is the longer generation intervals of wheat breeding and consequently the fewer number of generations represented. The results presented here show that incorporating more breeding cohorts in the training set is beneficial in a conventional breeding program with a long generation interval. A recent study by Gorjanc *et al.* (2018) investigates the response in a rapid cycling program which uses genomic selection to quickly identify parents.

While grain yield undergoes continual and intense selection within the breeding program, relative maturity and TKW are threshold traits and therefore change less over time, which results in them benefiting less from the inclusion of additional cohort years in the training set. Glaucousness undergoes no direct selection meaning genetic change will only occur through correlated response, and it therefore sees little benefit from adding more cohort years to the training set.

### Marker density

The effect of marker density on prediction accuracy was assessed with a random five-fold cross validation analysis performed with various marker densities. All traits experienced a strong response to increases in marker density up to 5,000 markers, showing that this was sufficient for generating a relatively accurate prediction calibration within this panel. This number is significantly higher than the plateau point of previous studies in smaller populations (Heffner *et al.*, 2011b), as high marker densities only facilitate finer resolution and more accurate estimates of QTL effects when combined with large population size and low linkage disequilibrium (Huang *et al.*, 2012). TKW benefited from increased marker density more than the other traits, which could be explained by its quantitative genetic nature requiring more markers to accurately estimate its many small QTL effects (Zhang *et al.*, 2015). However, grain yield is also a highly quantitative trait and it saw a similar response curve to the more qualitative traits glaucousness and relative maturity.

The interactive effect of marker density and population structure on prediction accuracy was assessed by repeating the K-means cluster analysis with various marker densities. The density at which prediction accuracy plateaued was slightly lower than that observed in the random five-fold cross validation. This is consistent with previous studies using smaller data sets where additional markers benefited prediction accuracy more when larger training sets were used (Heffner *et al.*, 2011a,b). Prediction accuracy responded more to increased marker density when predicting between clusters, particularly when there was more genetic diversity in the training set. This is consistent with the findings of Hickey *et al.* (2014), where in a simulated maize data set the required marker density was lower when closely related material was shared between training and validation sets. The study also showed there was greater response to increased marker density when the training set contained more diversity, which corroborates our findings. The slight decrease in prediction accuracy at high marker densities for relative maturity is likely due to excess markers overfitting the model (Heslot *et al.*, 2012). A similar result was seen in Heffner *et al.* (2011a), where higher marker densities resulted in lower prediction accuracy in bi-parental wheat populations.

### Conclusions

Here we used a wheat panel of unprecedented size to investigate several key factors affecting genomic prediction accuracy that previously have not been explored at this scale. We showed there is a point at which prediction accuracy begins to plateau in response to training set size, and that this response is independent from the genetic complexity of the trait. The population structure analyses showed that relatedness between training and validation sets has a large effect on prediction accuracy, but importantly when relatedness is low, as is often the case when applying genomic selection, prediction accuracy can be increased by increasing diversity in the training set. We also found that traits under higher selection pressure can be more accurately predicted by including several previous breeding cohorts in the training set. This was shown for up to three previous cohorts, but further work should be done to explore how stable this trend is across different breeding programs and more cohorts. By assessing the interaction between marker density and population structure, we showed the response to increased marker density is larger when using a diverse training set and predicting from poorly related training sets. The work presented herein provides a framework for pragmatic plant breeders to optimally design their genomic selection training strategy to achieve high selection accuracy and subsequent rates of genetic gain.
